# Impact of Surface Microstructure and Properties of Aluminum Electrodes on the Plating/Stripping Behavior of Aluminum-Based Batteries Using Imidazolium-Based Electrolyte

**DOI:** 10.1021/acsami.4c18168

**Published:** 2024-11-16

**Authors:** Ghadir Razaz, Irmgard Weißensteiner, Jonas Örtegren, Bernhard Trink, Stefan Pogatscher, Shahrzad Arshadi Rastabi

**Affiliations:** † Department of Engineering, Mathematics, and Science Education, Mid Sweden University, Holmgatan 10, 85170 Sundsvall, Sweden; ‡ Christian Doppler Laboratory for Advanced Aluminum Alloys, Chair of Nonferrous Metallurgy, Montanuniversität Leoben, Franz-Josef Straße 18, 8700 Leoben, Austria

**Keywords:** aluminum-based batteries (AIBs), Al 1 percent Fe, plating/stripping, surface microstructure, interphase layer

## Abstract

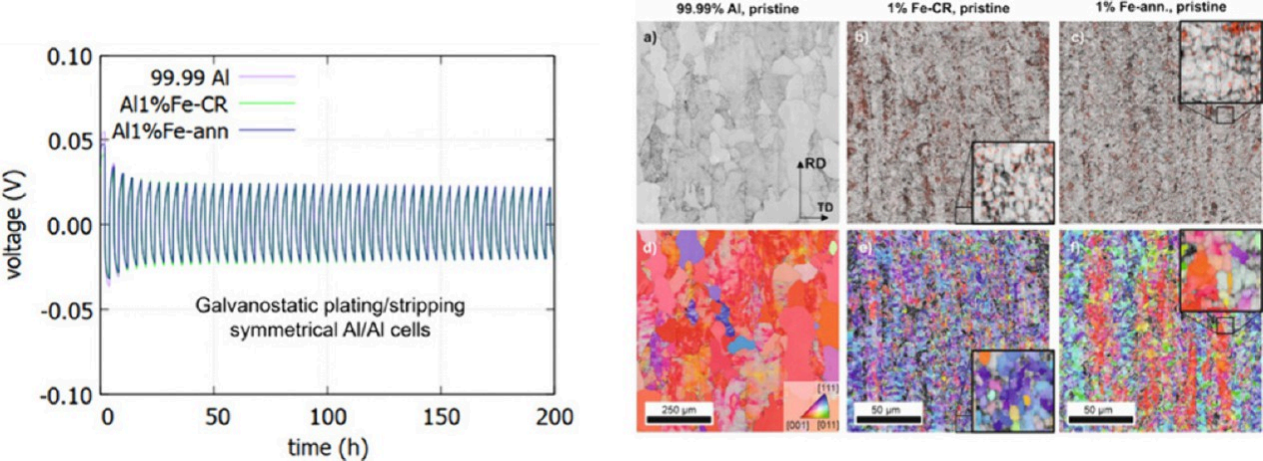

The 99.99% Al used for negative Al electrodes in aluminum-based battery studies is expensive. This is primarily due to the complex challenges associated with fabricating 99.99% Al, particularly the removal of Fe impurities from Al melts. Despite the importance of this issue for the future commercialization of Al-based batteries, it has been largely overlooked. This work accordingly studied the plating/stripping behavior of Al containing 1 wt % iron (Al 1% Fe) as an alternative electrode using conventional ([EMIm]Cl and AlCl_3_) electrolyte. Simultaneously, the impact of the surface microstructure of Al 1% Fe on the plating/stripping behavior was examined. The results indicate that the difference in the plating/stripping cycling of Al 1% Fe alloys and 99.99% Al is negligible. Thus, Al 1% Fe negative electrodes could serve as an efficient and commercially viable alternative to 99.99% Al for plating/stripping in Al-based batteries. This is an essential finding because facile and commercial fabrication of Al 1% Fe electrodes is absolutely feasible. The results are further discussed in terms of the impact of the Al surface microstructure (i.e., grain size, defect density, grain boundary distribution, crystal orientation, and intermetallic phases) on plating/stripping behavior. Moreover, this study provides insights into how the interphase layer formed on Al electrodes influences plating/stripping behavior.

## 1 Introduction

Currently, lithium-ion batteries (LIBs) dominate the battery market. However, LIBs face safety challenges and limited Li supplies run counter to sustainability requirements. This necessitates research into developing next-generation batteries with more sustainable characteristics.^1−5^ Among these batteries, aluminum (Al)-based batteries stand out as an alternative that is attractive from the sustainability perspective due to high Al abundance, good safety, ease of recycling, and low price. Besides, the ability for the multivalent-ion transfer of Al^3+^ could provide high theoretical gravimetric capacity and volumetric capacity with values of 2980 mAh g^–1^ and 8040 mAh cm^–3^, respectively. The above description places Al in an outstanding position compared with other metals for postlithium batteries.^6−10^ Intensive research into cathode materials for aluminum-based batteries has explored, for example, carbonaceous, transition-metal, and sulfur-based cathodes.^1,2,8^ As well, considerable research has been carried out to design and optimize both aqueous and nonaqueous electrolytes.^6,11,12^ A mixture of aluminum chloride and 1-ethyl-3 methylimidazolium chloride (AlCl_3_/[EMIm]Cl) is an established nonaqueous electrolyte for rechargeable Al batteries. This electrolyte enables the reversible plating/stripping of aluminum through the conversion between AlCl_4_^–^ and Al_2_Cl_7_^–^ species using various classes of cathode materials.^11,13^ Although considerable progress has been achieved in Al-based batteries, challenges remain to be addressed. Particularly when it comes to the Al negative electrode side, there are issues such as insulating oxide film, dendrite formation, volume expansion, and self-corrosion.^9,10,14^ Despite those issues, little attention has been paid to the Al negative electrode side. To develop Al-based batteries, it is therefore crucial to further explore their Al side. For instance, some works have studied various surface treatments and surface properties of Al and their impact on Al-based batteries. These treatments include mechanical polishing, electrochemical polishing, chemical etching, and the manufacturing process (e.g., rolling operations).^10,15−17^ One strategy for advancing Al negative electrodes is alloying research involving small additions of alloying elements to Al melts to produce Al alloy negative electrodes. With this strategy, the microstructure of Al negative electrodes, including the grain size, crystal orientation, and intermetallic phases, is altered and the surface properties of Al are thereby modified.^9^ This addresses issues related to Al alloy negative electrodes for Al-based batteries, such as passivating oxide film, dendrite formation, volume expansion, and self-corrosion.^9,10^ The technique of alloying Al is a convenient but insufficiently studied alternative for the development of rechargeable Al-based batteries. In previous works,^18,19^ we partly explored the impact of using negative electrodes of Al alloys with small iron contents in both nonaqueous and aqueous electrolytes. The results^18^ indicated that replacing high-purity Al (99.99%) negative electrodes with Al containing 1 wt % iron (Al 1% Fe) could enhance the cycling behavior of Al–graphite batteries using (AlCl_3_/[EMIm]Cl) electrolyte. It was explained that the Al_3_Fe phases forming in Al 1% Fe alloy could facilitate the fragmentation of the oxide film in contact with the electrolyte. Moreover, as discussed elsewhere,^13,14^ the high cost of [EMIm]Cl in well-established nonaqueous electrolytes for Al-based batteries is an obstacle to the development of Al-based batteries. There is a similar scenario for the typical pure Al (99.99%) negative electrode material used in rechargeable Al-based batteries, which is expensive because purifying the Al melt of Fe impurities to achieve 99.99% Al is a complicated procedure.^20^ Thus, using 99.99% Al as an electrode material in Al-based batteries absolutely prevents the development of such batteries, and a low-cost commercial Al alloy is needed as a substitute. In addition, from the mechanical property perspective, 99.99% Al has very poor strength, which could be another weakness of the use of 99.99% Al electrodes in Al-based batteries. While having a small Fe content of 1 wt % in Al alloy significantly improves the Al strength, it should also be noted that Fe is the main impurity found in Al melts. Due to its very low solubility (0.04 wt %) in Al melts, Fe will be precipitated as Al_3_Fe in the Al matrix.^21−24^

This work investigates, in detail, the stripping/plating behavior of Al 1% Fe in nonaqueous electrolyte, i.e., (AlCl_3_/[EMIm]Cl), exploring its potential as a low-cost alternative to the conventional 99.99% Al negative electrode material in Al-based batteries. Moreover, it was of great interest to analyze the effect of the surface modification of Al electrodes as an important factor influencing the stripping/plating behavior.^9,15,25,26^ Thus, two Al 1% Fe negative electrodes have been fabricated using different surface finishing processes, i.e., cold-rolling and annealing. The annealed and cold-rolled electrodes will have different microstructures and thus surface properties.^24^ In addition, this study produced considerable data on the formation of the interphase on the Al side, which is a parameter that could significantly influence plating/stripping performance.^27−30^

## 2 Experimental Procedures

### 2.1 Materials

Three types of aluminum alloy foils were used as negative electrodes in this work: 99.99% Al (purchased from Sigma-Aldrich, 130 μm thick), Al 1% Fe (as cold-rolled, 130 μm thick), and Al 1% Fe (annealed, 130 μm thick). Both the cold-rolled and annealed Al 1% Fe foils were produced at Montanuniversität Leoben, Austria.

#### 2.2.1 Al Electrode Fabrication

The Al 1% Fe alloys were melted at 750 °C in an MC100 V induction furnace (Indutherm, Walzbachtal Erwährmungsanlagen GmbH, Germany) and cast in a rectangular copper mold (cooling rate ≈60 °C s^–1^, capacity 100 g). The resulting ingots were homogenized at 500 °C for 1 h followed by air cooling. The ingots were hot rolled at 560 °C in a laboratory rolling mill, reducing their thickness from 12 to 7.3 mm. After that, the strips were cold rolled to a sheet thickness of 1 mm. The rolled material was then intermediately annealed at 560 °C for 30 min; subsequently, the strips were cold rolled to form 130-μm-thick foils. For one series of Al 1% Fe foils, a final soft annealing was performed at 560 °C for 30 min (Al 1% Fe-ann), whereas the other series of foils was fabricated just as cold rolled (Al 1% Fe-CR) to allow later study of the impact of Al foil surface microstructure on the stripping/plating process. The Al 1% Fe-CR and Al 1% Fe-ann foils, while identical in composition, therefore exhibit different microstructures—differing, for example, in grain size, defect density, grain boundary distribution, and orientation—allowing for comparative analysis of their effects on the stripping/plating behavior.

### 2.2 Electrochemical Measurements

The symmetrical Al alloy/Al alloy cells were assembled using 2032 coin-type cells with an ionic liquid electrolyte ([EMIm]Cl and AlCl_3_ in a 1:1.5 ratio; io-li-tec) in an argon-filled glovebox. Grade GF/D glass microfiber sheets (Whatman, Maidstone, UK) were used as separators. Also, polymeric separators (0.65-μm Durapore DVPP membrane filters; MilliporeSigma, Burlington, MA, USA) were applied to protect the Al surface in a few experiments. A schematic image of the symmetrical Al/Al cells is presented in Supporting Information Figure S1. The assembled cells were held for 3 h before starting the electrochemical tests. Cyclic voltammetry (CV) tests were carried out using a VersaSTAT 4 potentiostat (AMETEK, Berwyn, PA, USA) in a voltage range of −0.3 to 0.3 V at a scan rate of 5 mV s^–1^, as shown in Figure S2. Galvanostatic stripping/plating measurements were carried out at various current densities with a limiting capacity of 0.2 mAh cm^–2^. The electrochemical impedance spectroscopy measurements were carried out at an amplitude of 10 mV in the frequency range of 100 kHz to 100 mHz.

### 2.3 Material Characterization

The surface morphology and microstructure of the Al alloy electrodes were investigated using an MAIA3 field-emission scanning electron microscope (TESCAN, Brno, Czech Republic) at 15 kV. Energy-dispersive X-ray spectroscopy (EDX) (Oxford Instruments, Abingdon, U.K.) was also used to identify the chemical composition of the particles. Electron backscatter diffraction (EBSD) was used to examine the grain morphologies and sizes and was carried out on a JEOL 7200F field-emission scanning electron microscope (JEOL, Tokyo, Japan), equipped with an Oxford Symmetry S2 detector and an UltimMax100 EDS detector (Oxford Instruments). The acceleration voltage was set to 15 kV and the beam current to a value of 16. To remove deformed surface layers for EBSD analyses, the rolled material was electropolished with A2 electrolyte from Struers (Ballerup, Denmark) at a temperature of 5 °C and a voltage of 38 V for 18 s. For data analysis, Aztec Crystal software, version 3.1, and the mtex toolbox^31,32^ were used. X-ray photoelectron spectroscopy (XPS) measurements were performed on PHI 5500 (PHI, Chanhassen, MN, USA) and Kratos Axis Supra+ (Kratos, Manchester, U.K.) spectrometers equipped with monochromatic Al Kα radiation to analyze the interphase layers formed on cycled Al electrodes. The topographical images and roughness of the Al electrode surfaces were recorded using atomic force microscopy (AFM) (Park Systems, Suwon, South Korea). The AFM characterization was carried out in noncontact mode and the images were acquired at a scan rate of 1 Hz. For any post-mortem characterization, cells were disassembled in the argon-filled glovebox; Al electrodes were then soaked in DMC solvent (Sigma-Aldrich, Burlington, MA, USA) for 2 min to wash away the excess electrolyte.

## 3 Results and Discussion

### 3.1 Electrochemical Characterization

**Figure 1:**
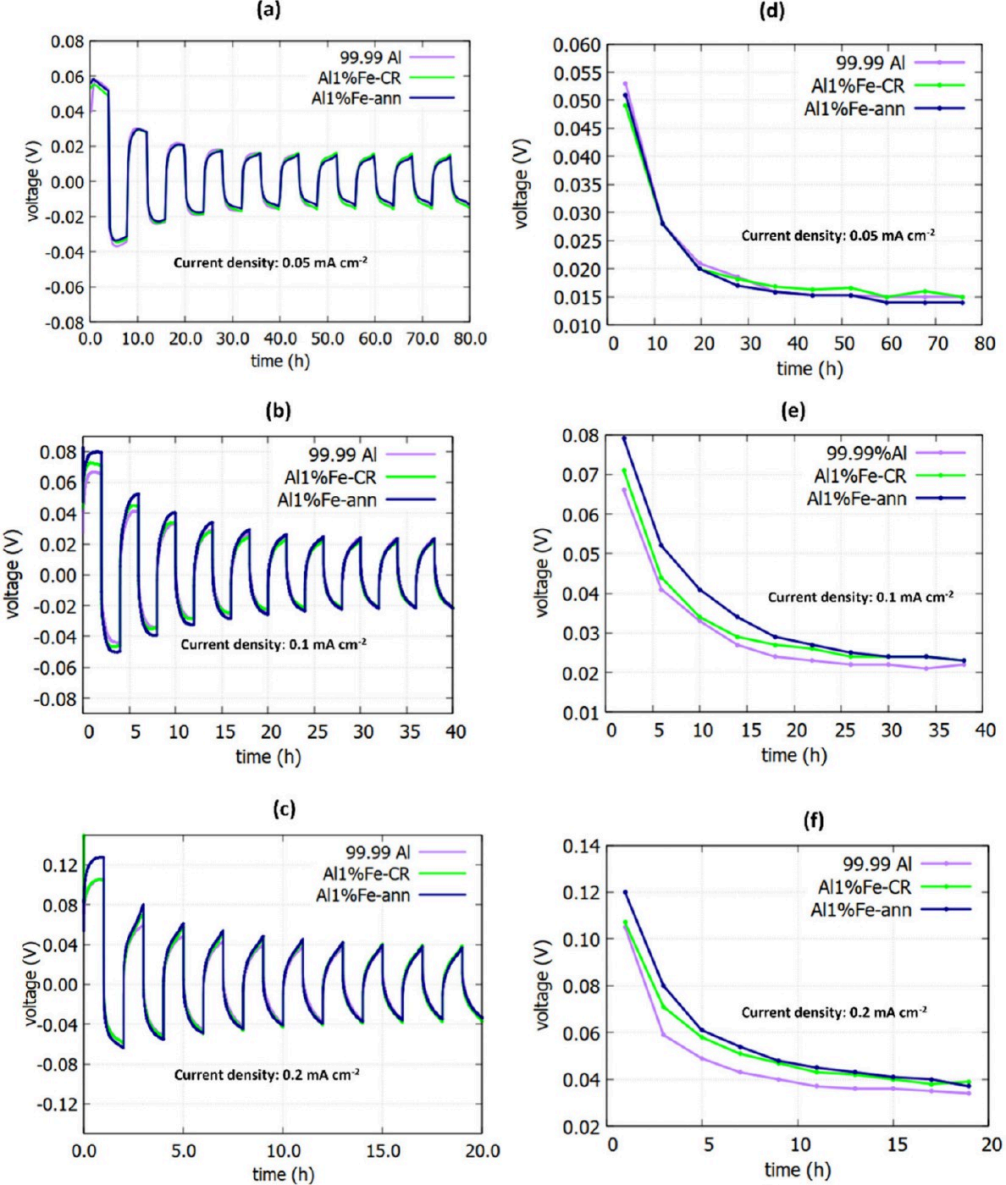
Galvanostatic plating/stripping profiles of symmetrical Al/Al cells constructed of different Al alloys. Voltage evolution for plating/stripping at a fixed limiting capacity of 0.2 mAh cm^–2^ and current densities of (a) 0.05 mA cm^–2^, (b) 0.1 mA cm^–2^, and (c) 0.2 mA cm^–2^. Corresponding voltage hysteresis profiles of ten positive plating/stripping curves for various symmetrical Al/Al cells at current densities of (d) 0.05 mA cm^–2^, (e) 0.1 mA cm^–2^, and (f) 0.2 mA cm^–2^.

**Figure 2:**
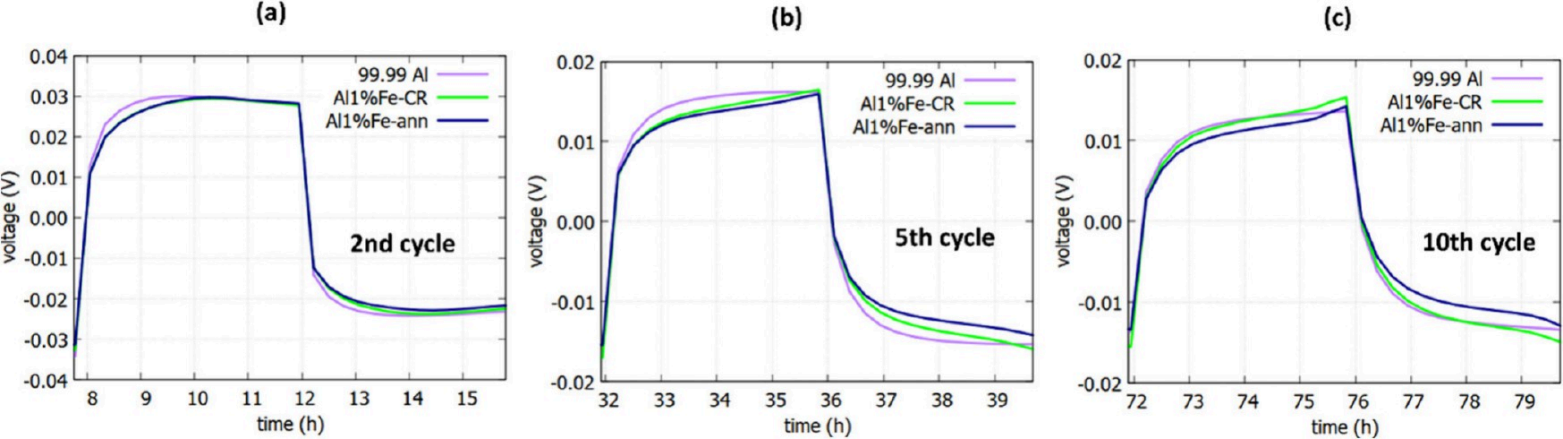
Detailed voltage profiles for plating/stripping at a current density of 0.05 mA cm^–2^ in Figure 1a: (a) second, (b) fifth, and (c) 10th cycles.

**Figure 3:**
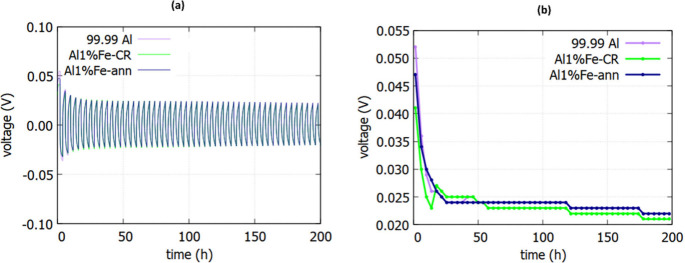
Galvanostatic plating/stripping profiles of symmetrical Al/Al cells constructed of different Al alloys: (a) voltage evolution over 200 h at a current density of 0.1 mA cm^–2^ and a limiting capacity of 0.2 mAh cm^–2^; (b) comparison of voltage hysteresis profiles for the plating/stripping of various symmetrical Al/Al cells.

A schematic image of Al plating/stripping in a symmetrical Al cell using ionic electrolyte is exhibited in Figure S3. It has been demonstrated that an appropriate concentration of the electroactive chloroaluminate anion, i.e., Al_2_Cl_7_^–^, in an ionic electrolyte, i.e., AlCl_3_/[EMIm]Cl = 1.5:1, could partly break down the insulating oxide film, facilitating the electrochemical activity.^28,30,33^ In the following, the stripping/plating behavior of symmetrical Al/Al cells using various Al alloys in an ionic liquid is investigated. Panels a–c of Figure 1 show the galvanostatic plating/stripping profiles of symmetrical Al alloy cells at various current densities with a limiting capacity of 0.2 mAh cm^–2^. It can be observed that 99.99% Al, Al 1% Fe-CR and Al 1% Fe-ann displayed similar trends in plating/stripping behavior. Panels d–f of Figure 1 show the corresponding voltage hysteresis profiles for the Al alloy plating/stripping shown in Figure 1a–c. At the lowest current density (0.05 mA cm^–2^), the voltage hysteresis profiles of various symmetrical Al alloy cells showed approximately the same values throughout each recorded cycle (as shown in Figure 1d). The initial overpotentials of all symmetrical cells were about 0.05 V, which flattened out and stabilized at a level of 0.017 V within about 30 h of cycling. In Figure 1e,f, starting from higher current densities of 0.1 and 0.2 mA cm^–2^, a similar trend in overpotential reduction can again be seen for all cells over the cycling. Over the first few cycles, the overpotentials of cells assembled from 99.99% Al were slightly lower than those of cells assembled from Al 1% Fe. Nevertheless, the overpotentials of all symmetrical cells approached one another over the cycling. It can be seen from Figure 1e that the difference in overpotential was negligible after about 30 h. For instance, the overpotential difference between symmetrical cells of 99.99% Al and Al 1% Fe after 30 h of cycling and at a current density of 0.1 mA cm^–2^ was about 9%, and after about 38 h of cycling was approximately 0%. High overpotentials were evident in the first few cycles for all types of symmetrical cells (Figure 1) due to the presence of an ion-insulating Al_2_O_3_ oxide layer on the Al surface. This Al_2_O_3_ native oxide layer was initially robust and impeded efficient plating/stripping, causing significant overpotential.^29,30,34^ Furthermore, a declining overpotential trend was observed over the cycling until stabilizing after about 30 h at a current density of 0.05 mA cm^–2^ (Figure 1d). In other words, in the first few cycles, high interfacial resistance was induced by the robust and ion-insulating oxide film on the Al surface. After that, oxide film gradually started breaking down in some locations in contact with the electrolyte. These defective sites in the Al oxide film allowed the electrolyte to access fresh Al surfaces, thus reducing the interfacial resistance. This explains why a dramatic decline in overpotential occurred within the first few cycles.^10,30,33,35^ It can be concluded that the Al surfaces were optimized within the first few cycles, displaying stable plating/stripping behavior through a trade-off area of robust and ruptured oxide film.^29,30,35^ The detailed voltage profiles for plating/stripping at a current density of 0.05 mA cm^–2^ for various cycles are illustrated in Figures 2a–c. As can be seen in Figure 2c, the plating curve in the stabilization position at the 10th cycle reveals a sharp tip for Al 1% Fe cells, which is absent for 99.99% Al cells. Figure 3a shows the plating/stripping profiles for a longer cycling time of 200 h at a current density of 0.1 mA cm^–2^ and a limiting capacity of 0.2 mA cm^–2^. The corresponding voltage hysteresis profiles in Figure 3b display a trend similar to that shown in Figure 1e. During the initial cycles, the overpotential values dropped substantially for various symmetrical Al cells. Afterward, at about 30 h of cycling, the overpotential values approached one another and stabilized. In the ensuing cycling up to 200 h, the overpotentials attained almost constant values.

**Figure 4:**
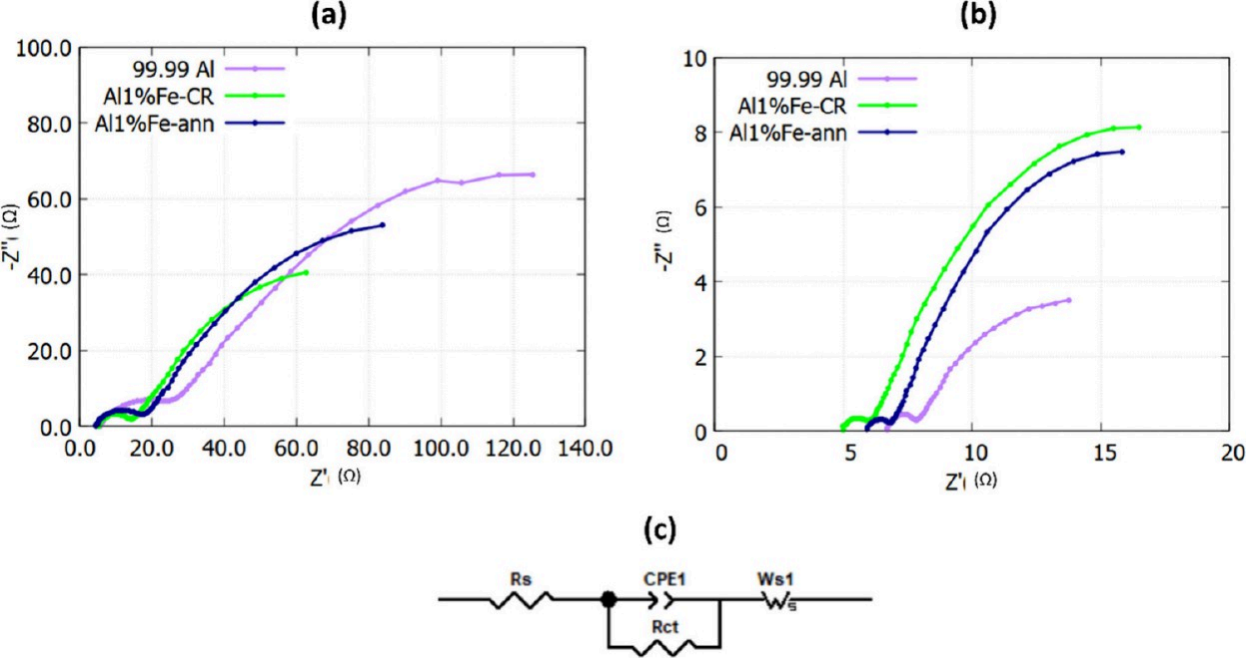
Nyquist plots from EIS measurements of various symmetrical Al/Al cells: (a) before cycling and (b) after 50 cycles. (c) Equivalent circuit model of the various symmetrical Al/Al cells.

In addition, electrochemical impedance spectroscopy was carried out for the symmetrical Al/Al cells shown in Figure 3, before cycling and after 50 cycles, as shown in Figure 4a,b. In the high-frequency region, the intercepts of the Nyquist plots with the real axis corresponded to the intrinsic resistance (*R*_s_) of both the electrolyte and electrode. All symmetrical cells displayed semicircular features in the high-middle-frequency range and inclined lines at the low frequencies before and after cycling. The diameters of the semicircular parts of the Nyquist plots in the high-frequency region were related to the interfacial resistance (*R*_ct_) of the electrolyte and electrode. The slope of the curves in the low-frequency region was representative of the Warburg diffusion impedance (*Z*_w_) and attributable to ion diffusion within the electrode.^13,28,30,36^Figure 4c shows the equivalent circuit model of the various symmetrical Al/Al cells, and the results of fitting the circuit components are given in Table 1. The *R*_s_ values for all symmetrical Al/Al cells reveal similar resistance before and after cycling. It can be calculated from Table 1 that the initial *R*_ct_ for a 99.99% Al cell was about 56% and 71% greater than for an Al 1% Fe-CR or Al 1% Fe-ann cell, respectively. This means that a symmetrical cell of 99.99% Al initially has a higher charge transfer resistance at the Al electrode/electrolyte interface. This is attributable to the existence of more robust native oxide film in 99.99% Al than in Al 1% Fe, which induces a higher interfacial resistance.^18,19,30^ However, the calculated *R*_ct_ values for 99.99% Al, Al 1% Fe-CR, and Al 1% Fe-ann symmetrical cells after cycling were 0.9, 0.8, and 0.7 Ω, respectively. This indicates a significant reduction in interfacial resistance for all symmetrical cells after cycling, in particular for 99.99% Al. The electrode/electrolyte interface resistance for all symmetrical Al cells eventually ended up at a similar value after cycling. It can be postulated that the native oxide film was either weakened or partly destroyed in contact with the electrolyte, which consequently reduced the interfacial resistance.^13,18,19,30,36^ This could also be evidenced by the result of the plating/stripping process, when the overpotential dropped dramatically in the initial cycling stages. The slopes of the Nyquist plots in the low-frequency region in Figure 4a indicate that the Warburg impedance (*Z*_w_) was similar for all symmetrical cells before cycling. However, after cycling, the slopes in the low-frequency region imply a lower *Z*_w_ for 99.99% Al than for Al 1% Fe cells. This suggests that there was higher resistance of ion diffusion into Al 1% Fe electrodes than into 99.99% Al electrodes during the cycling process.

**Table 1:** Fitted Value of the Circuit Components for Symmetrical Al/Al Cells before and after Cycling

	99.99% Al		Al 1% Fe-CR		Al 1% Fe-ann	
Fitted value of circuit components	before	after	before	after	before	after
*R*_s_ (Ω)	5.4	6.6	5.2	5	5	6
*R*_ct_ (Ω)	23.7	0.9	8	0.8	12	0.7

### 3.2 Material Analysis of Aluminum Alloy Electrodes

**Figure 5:**
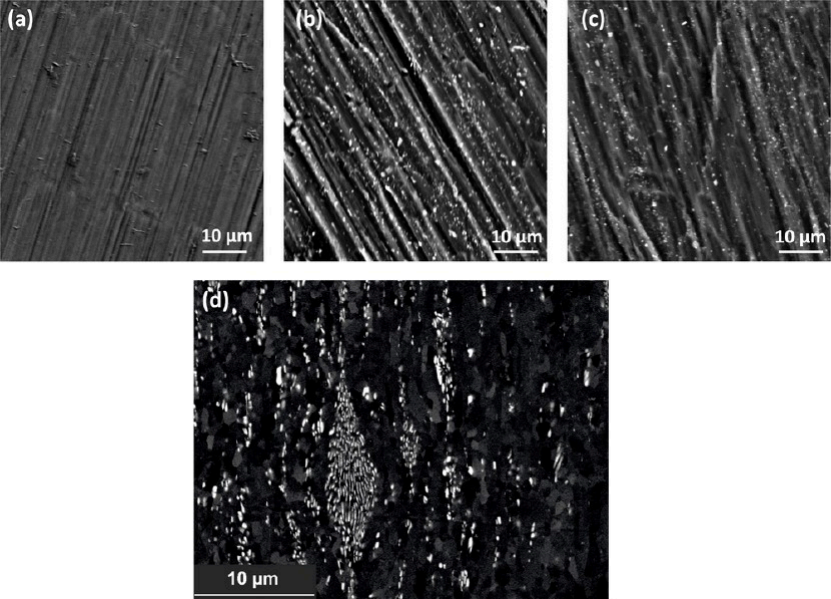
SEM secondary electron images of pristine Al alloy electrode surfaces: (a) 99.99% Al, (b) Al 1% Fe-CR, and (c) Al 1% Fe-ann. (d) Morphology of the Fe-rich intermetallic phases in CR condition from an electropolished sample.

Panels a–c of Figure 5 are SEM images of the pristine Al alloy electrode surfaces of 99.99% Al, Al 1% Fe-CR, and Al 1% Fe-ann. Due to the topography contrast of the secondary electrons, the rough rolled surface is visible. Both Al 1% Fe surfaces display numerous particles (bright dots) that are homogeneously dispersed in the Al matrix. The EDX analysis revealed that the composition of these particles corresponded to Al_3_Fe phases (Figure S4).^21,23,24^ Al_3_Fe particles were precipitated in the ≤1-μm size range. The altered chemical composition led to the precipitation of primary Al–Fe phases. In as-cast condition, these would represent sites of high chemical and structural inhomogeneity. However, if thermomechanically processed,^37^ they can be finely dispersed and used to design the microstructure of an Al negative electrode, including the grain size and crystal orientation. This allows for modification of the surface properties of Al. Figure 5d shows the chemical contrast of the Al 1% Fe alloy in the CR condition from an electropolished sample, with the bright phases being the Fe-rich intermetallic phases. Rolling operations fragmented the primary phases to fine particles of an average size of <1 μm. The dispersion was not completely homogeneous, which can be somewhat explained by the very soft Al matrix. Annealing changed neither the size nor morphology of the intermetallic particles.

**Figure 6:**
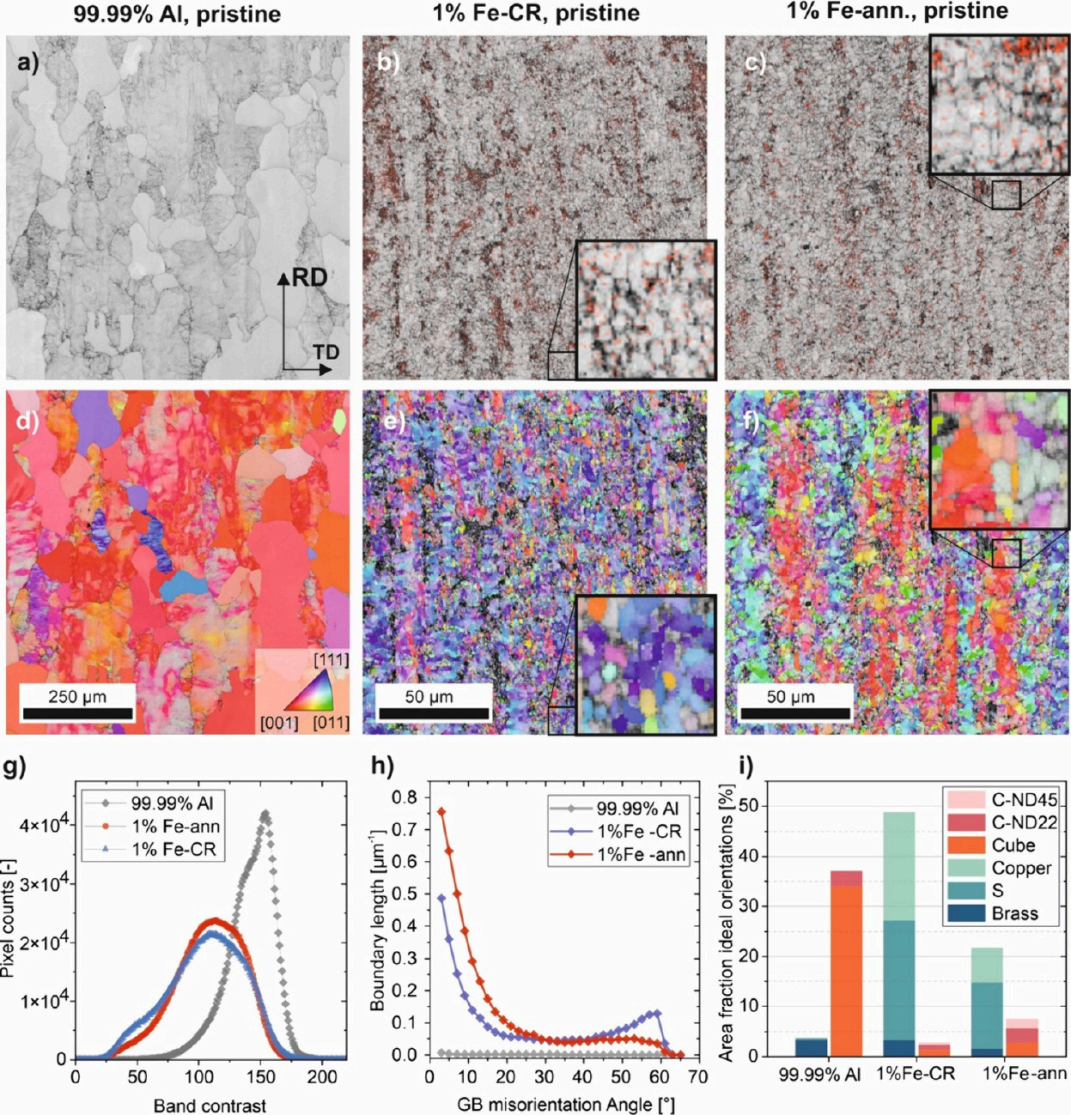
EBSD analyses of the three Al alloy conditions (note the different scale bars for 99.99% Al and Al 1% Fe): (a–c) band contrast depicting the microstructure of the materials, overlaid with the EDX Fe signal if a threshold that was exceeded at a data point; (d–f) inverse pole figure (IPF) maps after data cleanup (procedure described in ref (42)) (g) Histograms for the band contrast of all data points in the analyzed regions. (h) Histogram of misorientations in all detected orientations/grain boundary segments. (i) Area fractions of texture components (15° tolerance angle).

Figure 6 shows the microstructure of the material before testing, as determined from EBSD data. It is important to note that the analyses were performed near the surface, which might be less homogeneous or finer grained than the center. However, the primary objective was to characterize the type of microstructure exposed at the surface during the electrochemical tests. In Figure 6a–c, the structure is depicted via the band contrast (which can be correlated with defect density) and in Figure 6d–f via inverse pole figure (IPF) maps. Note the different magnification for the 99.99% Al sample. In these maps, the rolling direction is aligned vertically and the transverse direction horizontally. The color coding in Figure 6d–f represents the planes in the image plane, with no data rotation applied. The 99.99% Al material displays a significantly coarser structure, with grains that are clearly recrystallized and lack internal structures. However, some grains exhibit internal structures. Given that the material was electropolished, any deformation structures induced by sample preparation can be excluded. The IPF maps further confirm that the 99.99% Al material has undergone partial recrystallization, as indicated by the presence of orientation gradients visible as color gradients. The near-uniform red colors suggest a strong cubic texture ([001]⟨100⟩). In the band-contrast maps of Fe-containing alloys, both conditions display very fine microstructures. Fe-rich intermetallic particles are indicated by the EDX signal, with data points exceeding a specified threshold shown in red. As shown in Figure 5, these particles were very small. The differences between the conditions become more apparent when examining the grain orientations. In the CR condition, small well-defined globular subgrains with low in-grain misorientations are visible, which can be explained by the great tendency for the deposition of this material due to its high stacking fault energy, low solute content, and considerable strain during annealing.^38^ After annealing, the grains became coarser, with larger areas of similar orientations subdivided by low-angle grain boundaries (GBs). This could be a sign of discontinuous subgrain growth^38,39^ as the fraction of low-angle GBs increases, but the different coloring of the IPF maps suggests that variations in texture developed upon annealing, so partial recrystallization was more likely.^40,41^ The band-contrast histogram (Figure 6g) shows significantly higher values for the 99.99% Al material, reflecting a much lower density of GBs and the presence of recrystallized grains, in addition to those exhibiting deformation structures and orientation gradients. Together these two fractions form the gray sum curve of the histogram. In contrast, the curves for the cold-rolled (blue) and annealed (red) Al 1% Fe materials differ primarily in that the annealed curve lacks the shoulder observed in the sum curve. This could be explained by the fact that, upon annealing, the density of GBs decreased slightly (Figure 6h), primarily due to the reduction of high-angle GBs as a result of (sub)grain growth. Low-angle GBs persisted, likely due to the retarding effect of small intermetallic particles on dislocations. Texture analysis (Figure 6i) reveals a significant reduction in beta fiber orientations (comprising brass {011}⟨211⟩, copper {112}⟨111⟩, and S {123}⟨634⟩ orientation, tolerance angle: 12°) from over 50% to about 20% and a slight increase in Cube {001}⟨100⟩ and its rotated counterparts. As the material displayed significant recovery during cold rolling and fine Fe-rich particles were present, the material underwent only partial recrystallization during the annealing treatment. Subsequently, all these analyses imply that the surface microstructures of 99.99% Al, Al 1% Fe-CR, and Al 1% Fe-ann have very different grain sizes, defect densities, grain boundary distributions, and crystal orientations.

**Figure 7:**
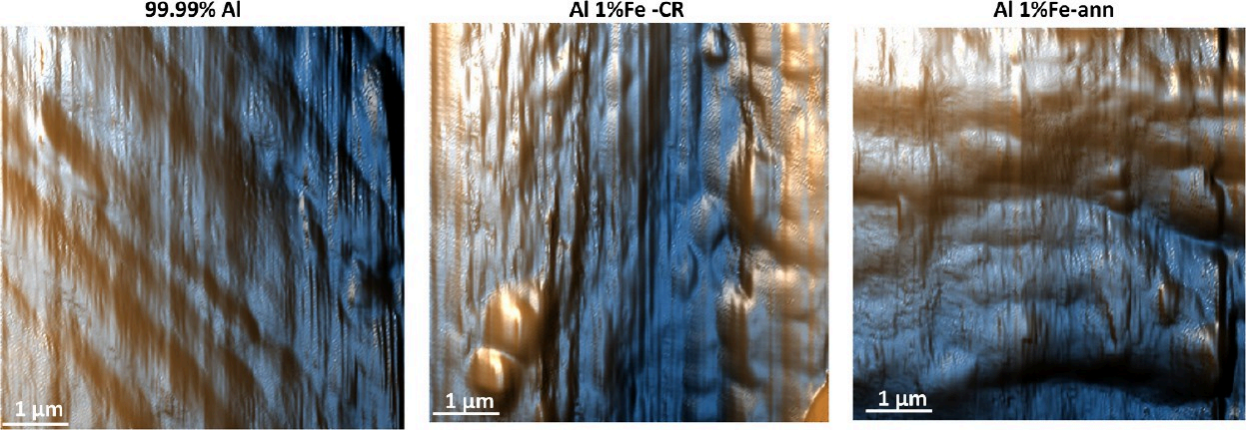
AFM surface topography of various pristine Al electrodes.

The final roughness of a rolled Al (or Al alloy) product can be significantly affected by the operation parameters or work roll roughness^43−45^ either by direct transfer of the roller roughness or via the formation of a disturbed surface layer. Figure 7 shows the AFM surface topography of different pristine Al electrodes. AFM analyses of five different spots (5 × 5 μm) has shown that roughness differed significantly among the Al alloys. The average roughness for 99.99% Al, Al 1% Fe-CR, and Al 1% Fe-ann electrodes was 873 ± 173, 487 ± 150, and 707 ± 207 nm, respectively. The 99.99% Al and the Al 1% Fe-CR material mainly exhibited longitudinal relief, likely stemming from the rolling mill. Particularly for the annealed condition of the Al–Fe material, another type of roughness was superimposed on the longitudinal pattern, possibly due to delamination of the disturbed surface layer during annealing, for example, due to the expansion of lubricant residues in/under this layer.

**Figure 8:**
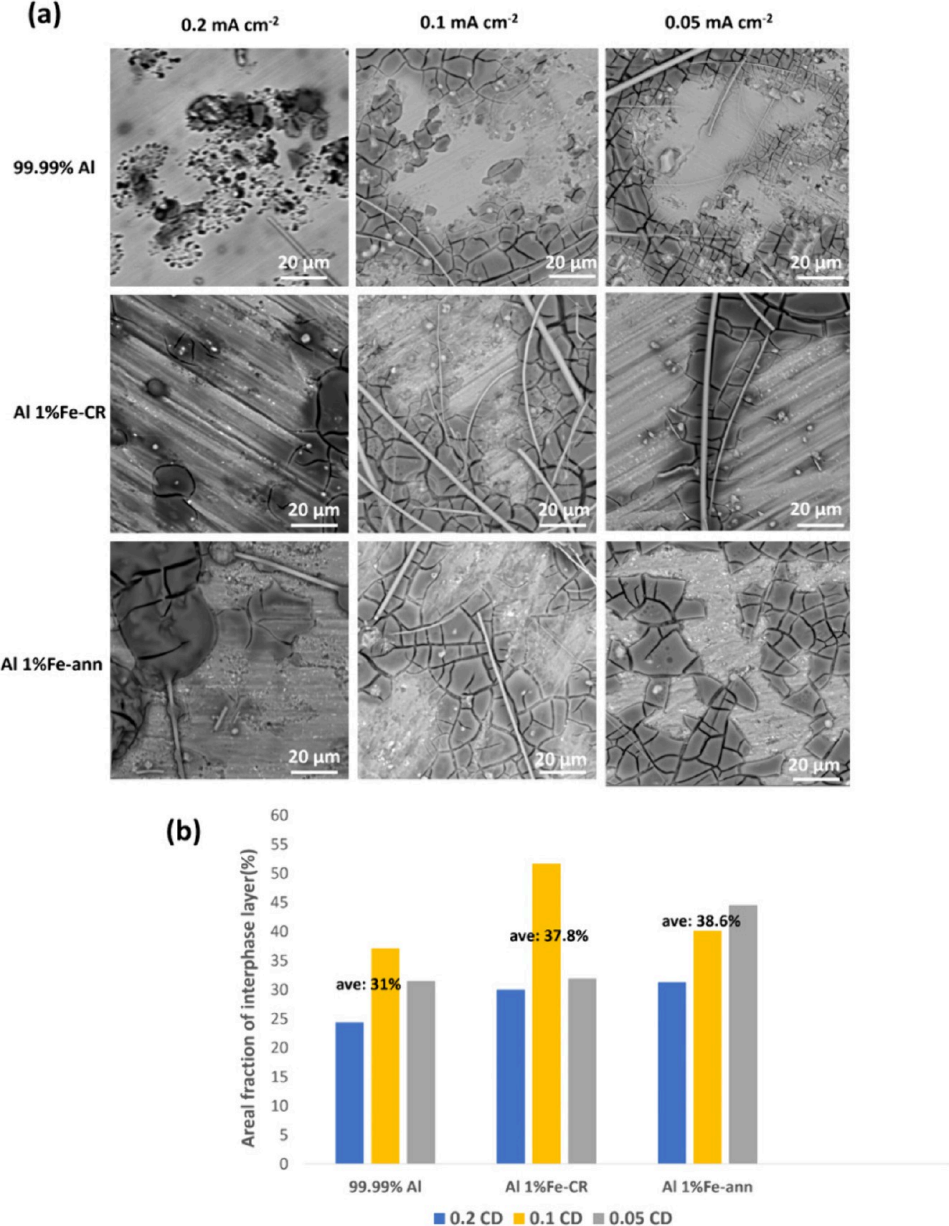
(a) Backscattered SEM images of different Al alloy electrodes from symmetrical Al/Al cells after ten cycles at various current densities (stripping/plating capacities were fixed at 0.2 mAh cm^–2^). (b) Corresponding fractional areas of interphase layers covering different Al electrode surfaces after ten plating/stripping cycles at various current densities (CD) at a fixed capacity of 0.2 mAh cm^–2^.

The interphase layer that forms on the electrode during electrochemical performance is a vital characteristic of the plating/stripping procedure, a characteristic that requires examination in more detail.^28,29^Figure 8a presents SEM images of the 99.99% Al, Al 1% Fe-CR, and Al 1% Fe-ann electrodes from the symmetrical Al/Al cells in Figure 1a–c after ten cycles of plating/stripping. The interphase layers on the Al alloy surfaces at different current densities displayed similar morphologies, as the mud structure contained cracks. Also, pieces of the glassy fiber separator (GF/D) could be observed in some locations. However, fewer cracks were seen at the highest current density of 0.2 mA cm^–2^ for all Al alloy electrodes, in particular for 99.99% Al. The area fractions of the interphase layer covering various Al alloys at different current densities were calculated using ImageJ image analyzer software and are shown in Figure 8b. The total analyzed area was 20 mm^2^, resulting from five 2 × 2 mm^2^ locations situated centrally in the electrodes. The average area fractions of the interphase layer for 99.99% Al, Al 1% Fe-CR, and Al 1% Fe-ann were 31.3%, 37.8%, and 38.6%, respectively, meaning that the interphase layer area fractions for Al 1% Fe-CR and Al 1% Fe-ann were 6.5% and 7.3% greater, respectively, than for 99.99% Al.

**Figure 9:**
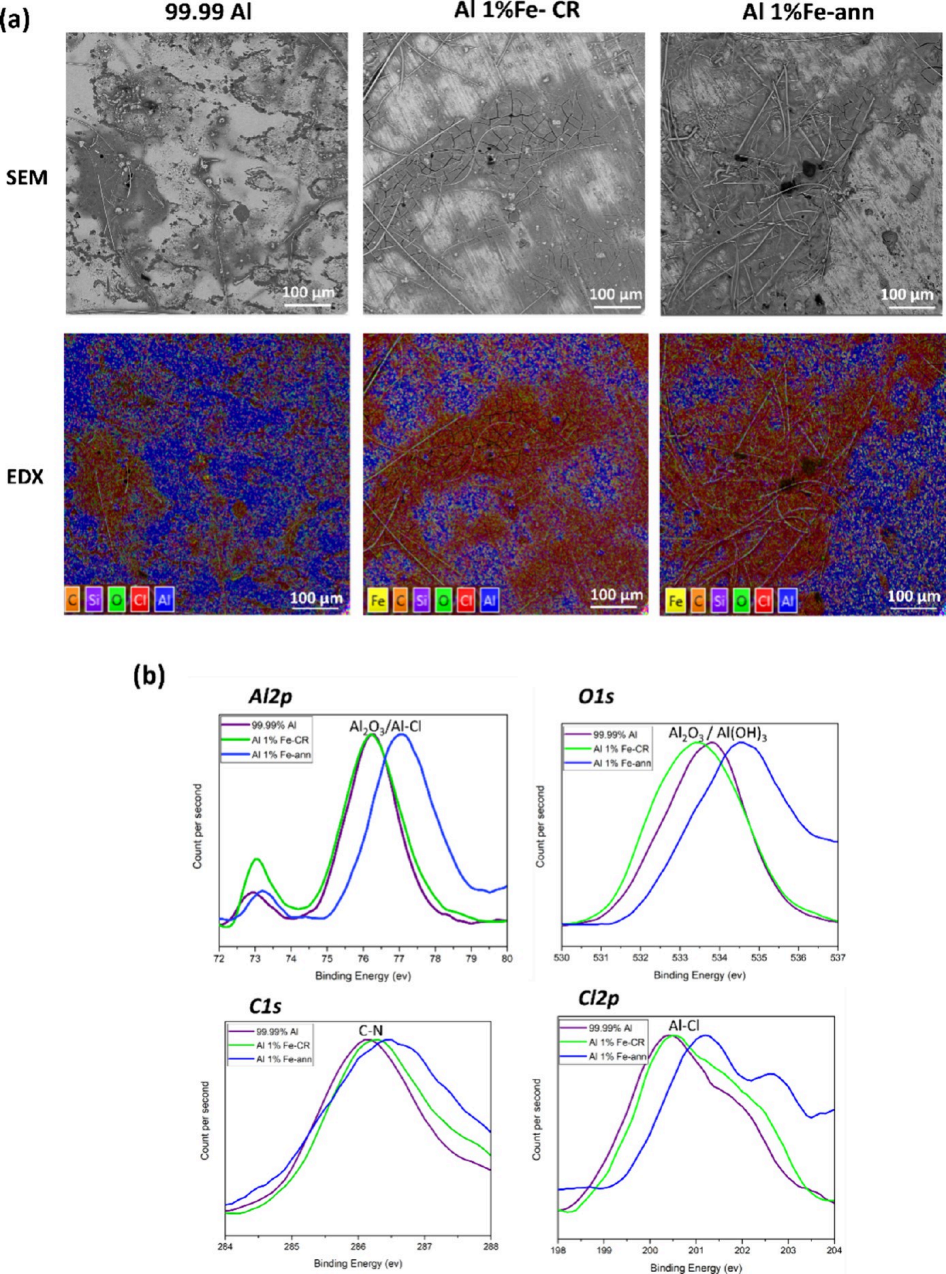
(a) Backscattered SEM images and corresponding EDX mapping analysis of Al electrodes after 50 cycles at a current density of 0.1 mA cm^–2^ with a plating/stripping capacity of 0.2 mAh cm^–2^. (b) Corresponding detailed XPS spectra of Al 2p, O 1s, C 1s, and Cl 2p from the interphase layer on Al electrodes after 50 cycles of plating/stripping.

Figures 9a and S5 are SEM and EDX analyses of 99.99% Al, Al 1% Fe-CR, and Al 1% Fe-ann electrodes from the symmetrical Al/Al cells shown in Figure 3 after 50 cycles of plating/stripping. It can be observed that the interphase layers on Al electrodes exhibited a morphology similar to that of the interphase layers shown in Figure 8a. EDX mapping of the cycled Al electrodes revealed Al, Cl, O, C, Fe, and Si elements. Figure S5 shows that Al, O, C, and Fe species were uniformly distributed on the surface, while Cl and Si species were mainly concentrated on the interphase layer and GF/D separator traces, corresponding to residual electrolyte and GF/D components, respectively.^35^

Furthermore, the interphase layer species on the Al electrode surfaces were analyzed using X-ray photoelectron microscopy (XPS) (Figure 9b). In the Al 2p spectra, two pairs of peaks can be seen. The first peak is observed at ∼73 eV for all Al alloys, which is corresponded to metallic Al. Whereas the second peak are seen at ∼76.2 eV for Al 1%Fe-CR and Al 1% Fe-ann that can be related to Al_2_O_3_ oxide and Al–Cl.^13,33,46^ However, such peak for Al 1% Fe-ann showed a ∼ 1 eV shift. O 1s spectra for 99.99% Al and Al 1% Fe-CR revealed a similar peak position at ∼533.2 eV that could be ascribed to either Al_2_O_3_ oxide or Al(OH)_3_ originating from corrosion products.^10,29^ However, O 1s spectra from Al 1% Fe-ann indicated a ∼ 2 eV shift. The C 1s peaks observed at ∼286.2 eV in can be assigned to the C–N bond. These peaks probably stemmed from decomposition species of ionic electrolyte containing imidazolium. The peaks observed in Cl 2p spectra for 99.99% Al and Al 1% Fe-CR revealed at ∼200.4 eV be attributed to Al–Cl species from AlCl_3_.^13,19,29^ However, a ∼ 1 eV shift for such Cl 2p spectra from Al 1% Fe-ann is seen. In addition, a clear extra peak from Cl 2p spectra for Al 1% Fe-ann is presented at ∼203 eV. It could be concluded that the species detected by XPS analysis were related to oxide film, corrosion products, and ionic electrolyte residue.

**Figure 10:**
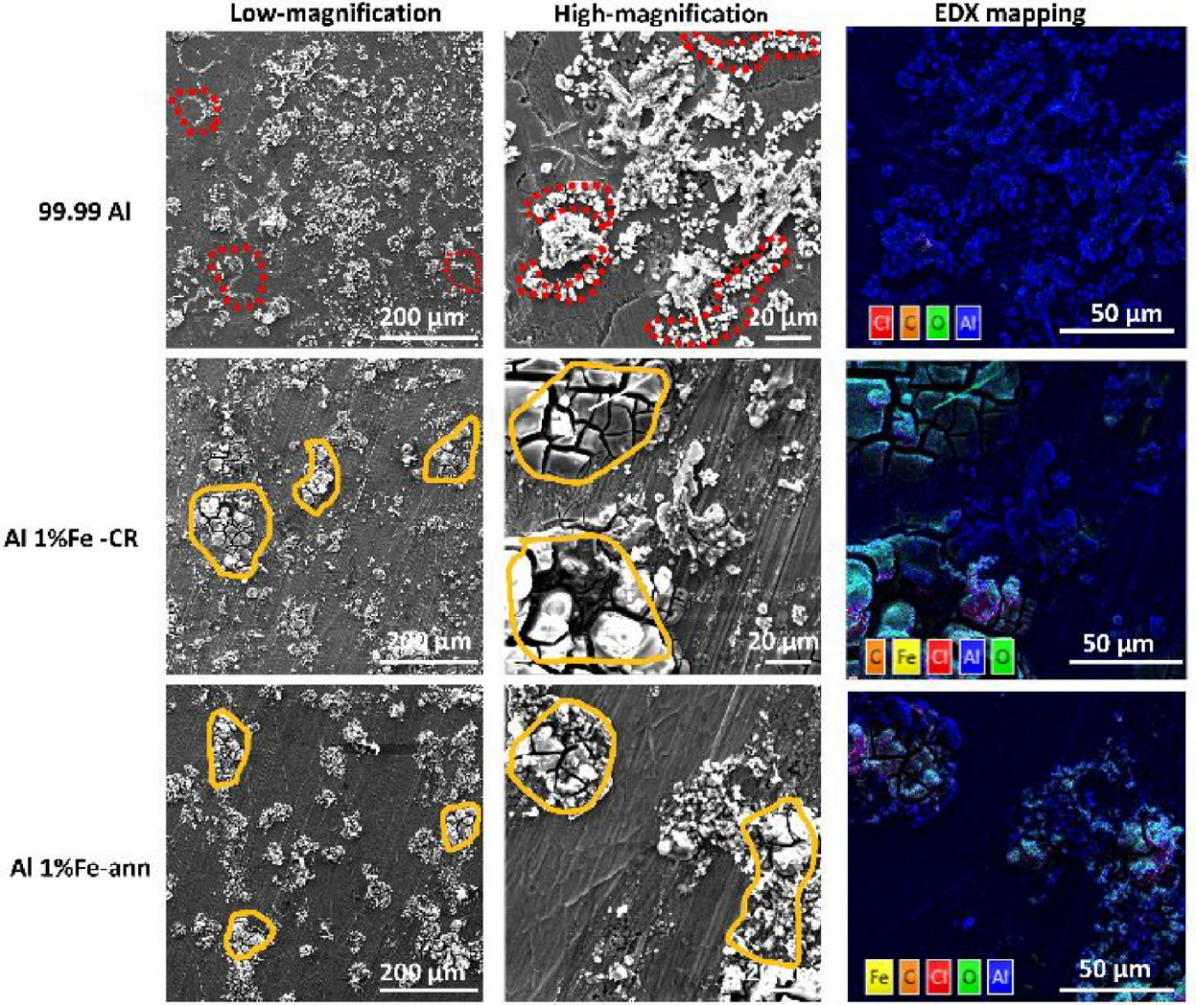
SEM secondary electron images and corresponding EDX mapping analyses of various Al electrodes at the first plating cycle at a current density of 0.05 mA cm^–2^ and fixed capacity of 0.5 mAh cm^–2^. The low- and high-magnification images of each electrode depict the same region. The red dashed areas show the Al tendency for deposition along the grain boundaries for 99.99% Al, and the yellow circled areas indicate the regions that byproducts are mainly accumulated in Al 1%Fe alloys.

To further explore the interphase layer, symmetrical Al/Al cells were constructed using ultrathin Durapore PVDF membrane (MilliporeSigma, Burlington, MA, USA) on the Al sides. This was done because the GF/D separator became partly stuck to the Al surface during cycling, and adding the PVDF membrane could protect the Al surface from this. These symmetrical Al/Al cells were run at a current density of 0.05 mA cm^–2^ and fixed capacity of 0.5 mAh cm^–2^. Next, the tests were stopped at the first plating cycle to characterize the plating phenomenon on the Al surface. Figure 10 shows the footprints of the Al plating phenomenon at the first cycle for the various Al electrodes. In 99.99% Al, the GBs in some regions are marked by Al deposition, indicating a tendency for Al to start depositing along the GBs. On the other hand, from Al 1% Fe, it can be observed that, even in an initial cycle, byproduct has already formed on the electrode surface. This can be related to the larger amount of byproduct coming from corrosion of the Al surface of Al 1% Fe, i.e., Al(OH),^29^ as was also shown in Figure 8b. Therefore, these byproducts are probably the cause of the slightly higher overpotential seen in Figures 1, 2, and 3 for symmetrical Al cells of Al 1% Fe compared with those of 99.99% Al. It has been observed^18,19^ that the oxide film on Al 1% Fe is more susceptible to being destroyed in contact with the electrolyte than is the film on 99.99% Al, due to its having intermetallic Al_3_Fe phases. That is why higher corrosion contents were created on Al 1% Fe electrodes during cycling.

## 4 Conclusions

The plating/stripping behavior of symmetrical Al/Al cells in ionic liquid electrolyte was analyzed focusing on 99.99% Al and Al containing 1 wt % iron (Al 1% Fe). The results indicated that Al 1% Fe has plating/stripping behavior similar to that of 99.99% Al in ionic liquid electrolyte for Al-based batteries. Thus, Al 1%Fe could be an alternative to expensive 99.99% Al. The results also indicated that surface microstructure variables, i.e., grain size, defect density, grain boundary distribution, and crystal orientation, do not affect plating/stripping performance. Similarly, surface properties such as roughness displayed no notable influence on the behavior of the symmetrical Al/Al cells. However, the presence of intermetallic phases was found to have a slight effect on the plating/stripping behavior, leading to the minor increase in overpotential observed in the Al 1% Fe symmetrical cells compared with the 99.99% Al cells. This increased overpotential is attributable to a larger interphase layer forming on the surface of Al 1% Fe electrodes, likely due to the higher corrosion product formation associated with the intermetallic phases in Al 1% Fe. These findings highlight the importance of considering intermetallic phases and interphase layer formation in the design and optimization of Al-based battery systems.

## Supporting Information Available

Schematic image of Al/Al symmetrical cell assembly; cyclic voltammetry tests of symmetrical Al/Al cells constructed of different Al alloys; EDX analysis of bright particles precipitated in pristine Al matrix; EDX mapping analysis of Al alloy electrodes after 50 cycles at 0.1 mA cm^–2^ fixed current density with 0.2 mAh cm^–2^ plating/stripping capacity (PDF)
