# Supplementary material for: Impact of Surface Microstructure and Properties of Aluminum Electrodes on the Plating/Stripping Behavior of Aluminum-Based Batteries Using Imidazolium-Based Electrolyte
Source: ACS Appl Mater Interfaces. 2024 Nov 16;16(47):65725–36. doi: 10.1021/acsami.4c18168 (PMC11615850; doi:10.1021/acsami.4c18168)
Supplement: Supplementary file 1 — am4c18168_si_001.pdf [file am4c18168_si_001.pdf]

Supporting Information

Impact of Surface Microstructure and Properties of Aluminum Electrodes on the Plating/Stripping Behavior of Aluminum-Based Batteries using Imidazolium- based Electrolyte

Ghadir Razaz,[*,a] *Irmgard Weißensteiner,*[b] *Jonas Örtegren,*[a] *Bernhard Trink,*[b] *Stefan Pogatscher,*[b] *and Shahrzad Arshadi Rastabi* [a]

[a] Department of Engineering, Mathematics, and Science Education, Mid Sweden University, Holmgatan 10, 85170 Sundsvall, Sweden

[b] Christian Doppler Laboratory for Advanced Aluminum Alloys, Chair of Nonferrous Metallurgy, Montanuniversität Leoben, Franz-Josef Straße 18, 8700 Leoben, Austria

Corresponding author: ghadir.razaz@miun.se

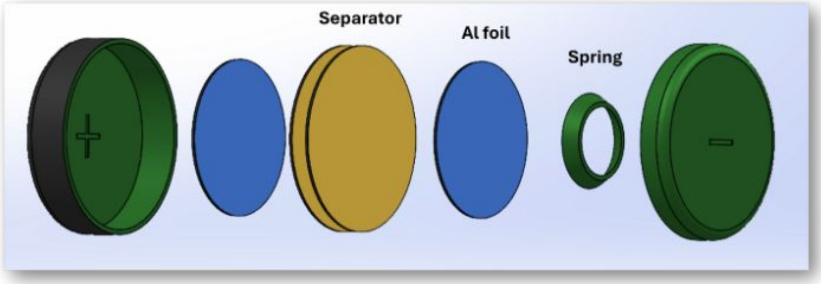

Figure S1. schematic picture of the Al/Al symmetric cell assembly.

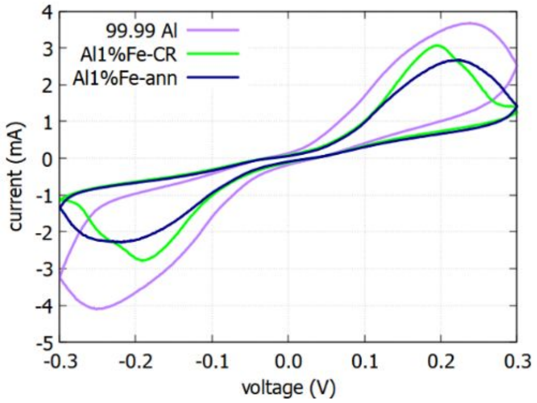

Figure S2. The Cyclic voltammetry (CV) tests symmetric Al/Al cells constructed of different Al alloys.

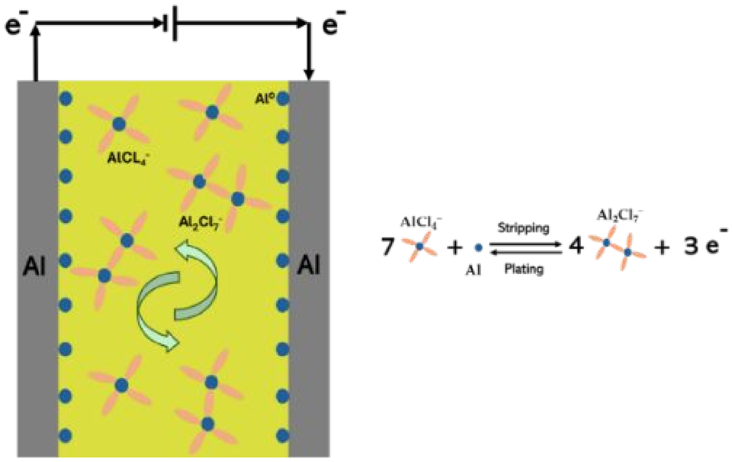

Figure S3. Al plating/stripping procedure for a symmetrical Al/Al cell in ionic liquid electrolyte, i.e., ($AlCl_3$/[EMIm]Cl) [1,2].

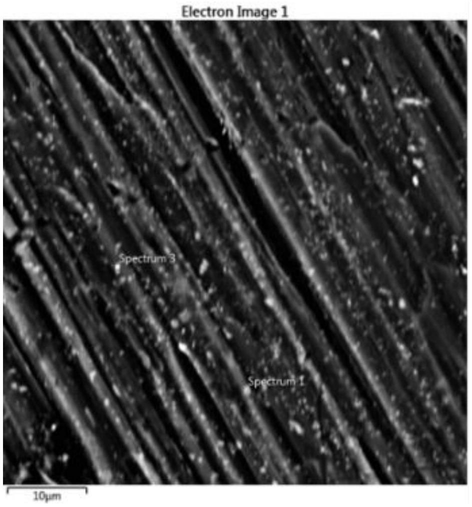

	Spectrum 1
Element	Weight % σ
Al	93.6
Fe	6.4

Figure S4. EDX analysis of bright particles precipitated in pristine Al matrix.

99.99 Al

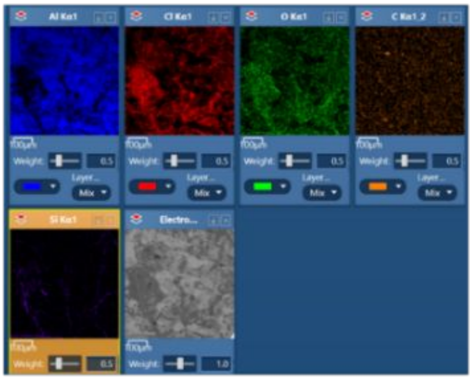

Map Sum Spectrum

Element	Weight %
Al	56.0
C	22.5
O	12.3
Cl	8.9
Si	0.3

Al 1%Fe- Cr

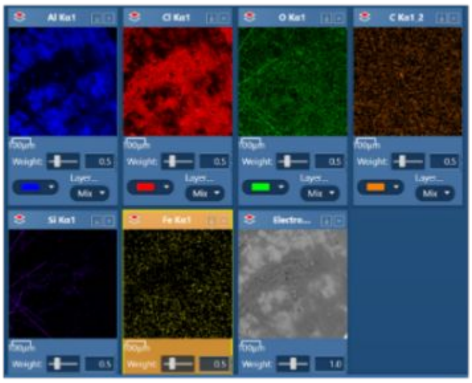

Map Sum Spectrum

Element	Weight %
Al	41.2
C	23.3
Cl	17.8
O	16.4
Si	0.7
Fe	0.7

Al 1%Fe- An

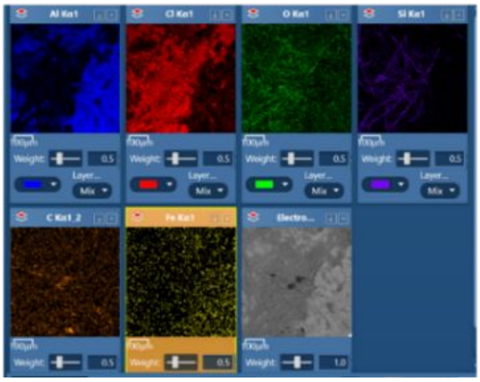

Map Sum Spectrum

Element	Weight %
Al	39.1
C	26.2
Cl	17.0
O	15.5
Si	1.6
Fe	0.5

Figure S5. EDX mapping analysis of Al alloy electrodes after 50 cycling at fixed current densities of 0.1 mA cm^{-2} with plating/stripping capacities of 0.2 mAh cm^{-2}. (discrete mapping images).

References:

1. Long Y, Li H, Ye M, Chen Z, Wang Z, Tao Y, Weng Z, Qiao SZ, Yang QH. Suppressing Al Dendrite Growth towards a Long-Life Al-metal Battery. Energy Storage Materials. 2021 Jan 1;34:194-202.

2. Long, Bo, Feng Wu, Yu Li, Haoyi Yang, Wenhao Liu, Ying Li, Qiaojun Li, Xin Feng, Ying Bai, and Chuan Wu. "Manipulating the Corrosion Homogeneity of Aluminum Anode toward Long-Life Rechargeable Aluminum Battery." Carbon Neutralization 3, no. 1 (2024): 64-73.
